# Supplementary material for: Peroxisomal lactate dehydrogenase is generated by translational readthrough in mammals
Source: eLife. 2014 Sep 23;3:e03640. doi: 10.7554/eLife.03640 (PMC4359377; doi:10.7554/eLife.03640)
Supplement: Supplementary file 1. — Plasmids used in this study. DOI: http://dx.doi.org/10.7554/eLife.03640.025 [file elife03640s001.docx]

**Supplementary file 1.** Plasmids used in this study.

| **Plasmid no. PST** | **Plasmid name / genotype** | **Source** |
| --- | --- | --- |
| 1360 | pDRVL | This study |
| 1384 | pDRVL-ZNF574 | This study |
| 1385 | pDRVL-LDHB | This study |
| 1387 | pDRVL-PPP1R3F | This study |
| 1393 | pDRVL-LDHB [TGG] | This study |
| 1394 | pDRVL-LDHB [TGAT] | This study |
| 1395 | pDRVL-LDHB [TAA] | This study |
| 1396 | pDRVL-LDHB [TAAT] | This study |
| 1418 | pDRVL-LENG1 | This study |
| 1419 | pDRVL-PRDM10 | This study |
| 1420 | pDRVL-FBXL20 | This study |
| 1421 | pDRVL-THG1L | This study |
| 1422 | pDRVL-EDEM3 | This study |
| 1423 | pDRVL-EDN1 | This study |
| 1424 | pDRVL-UBQLN1 | This study |
| 1425 | pDRVL-IRAK3 | This study |
| 1426 | pDRVL-SLC3A1 | This study |
| 1430 | pDRVL-LEPRE1 | This study |
| 1435 | pDRVL-MDH1 | This study |
| 1437 | pDRVL-VASN | This study |
| 1493 | pDRVL-AQP4 | This study |
| 1494 | pDRVL-SYTL2 | This study |
| 1497 | pDRVL-CACNA2D4 | This study |
| 1504 | pDRVL-DHX38 | This study |
| 1444 | pDRVL-VASN-doubleTAA | This study |
|  | pEYFP-C1 | Clontech |
|  | pECFP-C1 | Clontech |
|  | pOTB7-LDHB (clone HsCD00334443) | plasmID |
| 1388 | pEYFP-C1-LDHBx | This study |
| 1389 | pEYFP-C1-LDHBx[TGG] | This study |
| 1407 | pEYFP-C1-LDHBx[ΔL] | This study |
| 1408 | pEYFP-C1-LDHBx [SSI] | This study |
| 1409 | pEYFP-C1-LDHB [TGAT] | This study |
| 1410 | pEYFP-C1-LDHB [TAA] | This study |
| 1411 | pEYFP-C1-LDHB [TAAT] | This study |
| 1434 | pEYFP-C1-LDHA | This study |
| 1440 | pECFP-C1-LDHBx [TGG] | This study |
| 1512 | pECFP-C1-LDHBx [TGG, ΔL] | This study |
| 1513 | pECFP-C1-LDHBx [TGG, SSI] | This study |
|  | pENTR-TOPO-D | Invitrogen |
|  | pEXP-N-Venus | Ania Muntau lab |
|  | pEXP Venus-hRluc | Ania Muntau lab |
|  | pcDNA3.1/myc-His (-)A | Invitrogen |
| 1209 | pENTR-TOPO-D-PTS1 (ACOX3) | This study |
| 1226 | pEXP-N-Venus-PTS1 (ACOX3) | This study |
| 1441 | pcDNA3.1-HA-LDHBx-myc | This study |
| 1442 | pcDNA3.1-HA-LDHBx[TGG]-myc | This study |
| 1456 | pcDNA3.1-HA-LDHBx[TGA T]-myc | This study |
| 1457 | pcDNA3.1-HA-LDHBx[TAA]-myc | This study |
| 1458 | pcDNA3.1-HA-LDHBx[TAA T]-myc | This study |
