# Supplementary material for: Peroxisomal lactate dehydrogenase is generated by translational readthrough in mammals
Source: eLife. 2014 Sep 23;3:e03640. doi: 10.7554/eLife.03640 (PMC4359377; doi:10.7554/eLife.03640)
Supplement: Supplementary file 2. — Oligonucleotides used in this study. DOI: http://dx.doi.org/10.7554/eLife.03640.026 [file elife03640s002.docx]

**Supplementary file 2.** Oligonucleotides used in this study. DR. dual reporter.

| **OST No.** | **Name** | **Sequence 5’ – 3’** |
| --- | --- | --- |
| 801 | PTS1 (ACOX3) for | CACCCCTGTCATAGGAAGTCTGAAATCGAAGCTCTAG |
| 802 | PTS1 (ACOX3) rev | CTAGAGCTTCGATTTCAGACTTCCTATGACAGGGGTG |
| 963 | DR MCS for | TCGAGCGGTCACCATCGATTCCGGACCGTACGG |
| 964 | DR MCS rev | TCGACCGTACGGTCCGGAATCGATGGTGACCGC |
| 1053 | LDHB for | GCGCGAATTCTATGGCAACTCTTAAGGAAAAAC |
| 1054 | LDHB rev | GCGCTCTAGACTACAGCCTAGAGCTCAC |
| 1055 | LDHB [TGG] rev | GCGCTCTAGACTACAGCCTAGAGCTCACTAGCCACAGGTCTTTTAGG |
| 1081 | DR-ZNF574 for | GTCACCATATCAGTGGCTGACTCTGCCCGAT |
| 1082 | DR-ZNF574 rev | CCGGATCGGGCAGAGTCAGCCACTGATATG |
| 1083 | DR-LDHB for | GTCACCAAAAAGACCTGTGACTAGTGAGCTT |
| 1084 | DR-LDHB rev | CCGGAAGCTCACTAGTCACAGGTCTTTTTG |
| 1086 | DR-PPP1R3F for | GTCACCATTGGTTCTCATAGGCTCTGCTTGT |
| 1087 | DR-PPP1R3F rev | CCGGACAAGCAGTGCCTATGAGAACCAATG |
| 1117 | DR-LDHB [TGAT] for | GTCACCAAAAAGACCTGTGATTAGTGAGCTT |
| 1118 | DR-LDHB [TGAT] rev | CCGGAAGCTCACTAATCACAGGTCTTTTTG |
| 1119 | DR-LDHB [TAA] for | GTCACCAAAAAGACCTGTAACTAGTGAGCTT |
| 1120 | DR-LDHB [TAA] rev | CCGGAAGCTCACTAGTTACAGGTCTTTTTG |
| 1121 | DR-LDHB [TAAT] for | GTCACCAAAAAGACCTGTAATTAGTGAGCTT |
| 1122 | DR-LDHB [TAAT] rev | CCGGAAGCTCACTAATTACAGGTCTTTTTG |
| 1123 | DR-LDHB [TGGC] for | GTCACCAAAAAGACCTGTGGCTAGTGAGCTT |
| 1124 | DR-LDHB [TGGC] rev | CCGGAAGCTCACTAGCCACAGGTCTTTTTG |
| 1125 | LDHB [ΔL] rev | GCGCTCTAGACTACCTAGAGCTCACTAGTCAC |
| 1126 | LDHB [SSI] rev | GCGCTCTAGACTATATACTAGAGCTCACTAGTC |
| 1127 | LDHB [TAA] rev | GCGCTCTAGACTACAGCCTAGAGCTCACTAGTTACAGGTCTTTTAGG |
| 1128 | LDHB [TAAT] rev | GCGCTCTAGACTACAGCCTAGAGCTCACTAATTACAGGTCTTTTAGG |
| 1129 | LDHB [TGAT] rev | GCGCTCTAGACTACAGCCTAGAGCTCACTAATCACAGGTCTTTTAGG |
| 1263 | LDHB TGG [ΔL] rev | GCGCTCTAGACTACCTAGAGCTCACTAGCCAC |
| 1264 | LDHB TGG [SSI] rev | GCGCTCTAGACTATATACTAGAGCTCACTAGCCA |
| 1130 | LDHA for | GCGCGAATTCTATGGGTGAACCCTCAGGA |
| 1131 | LDHA rev | GCGCTCTAGATTAAAATTGCAGCTCCTTTTGG |
| 1144 | DR-LENG1 for | GTCACCGCCTTACTCACTGACTCCTGAGGGT |
| 1145 | DR-LENG1 rev | CCGGACCCTCAGGAGTCAGTGAGTAAGGCG |
| 1148 | DR-PRDM10 for | GTCACCGCACCAAACCATGACTTCCACCCTT |
| 1149 | DR-PRDM10 rev | CCGGAAGGGTGGAAGTCATGGTTTGGTGCG |
| 1150 | DR-FBXL20 for | GTCACCGCATCATCCTATGACAATGGAGGTT |
| 1151 | DR-FBXL20 rev | CCGGAACCTCCATTGTCATAGGATGATGCG |
| 1152 | DR-THG1L for | GTCACCGAGCCAGGCTTTGACGGAAGAGTCT |
| 1153 | DR-THG1L rev | CCGGAGACTCTTCCGTCAAAGCCTGGCTCG |
| 1154 | DR-EDEM3 for | GTCACCGGGATGAGCTATGACTTGCTAAACT |
| 1155 | DR-EDEM3 rev | CCGGAGTTTAGCAAGTCATAGCTCATCCCG |
| 1156 | DR-EDN1 for | GTCACCGAGCACATTGGTGACAGACCTTCGT |
| 1157 | DR-EDN1 rev | CCGGACGAAGGTCTGTCACCAATGTGCTCG |
| 1158 | DR-LEPRE1 for | GTCACCGGGATGAGCTATGACAGCGTCCAGT |
| 1159 | DR-LEPRE1 rev | CCGGACTGGACGCTGTCATAGCTCATCCCG |
| 1160 | DR-UBQLN1 for | GTCACCGCCAGCCATCATAGCAGCATTTCTT |
| 1161 | DR-UBQLN1 rev | CCGGAAGAAATGCTGCTATGATGGCTGGCG |
| 1162 | DR-IRAK3 for | GTCACCGCAAAAAAGAATAAATTCTACCAGT |
| 1163 | DR-IRAK3 rev | CCGGACTGGTAGAATTTATTCTTTTTTGCG |
| 1164 | DR-SLC3A1 for | GTCACCGTACCTCGTGTTAGGCACCTTTATT |
| 1165 | DR-SLC3A1 rev | CCGGAATAAAGGTGCCTAACACGAGGTACG |
| 1190 | DR-MDH1 for | GTCACCGTTCCTCTGCCTGACTAGACAATGT |
| 1191 | DR-MDH1 rev | CCGGACATTGTCTAGTCAGGCAGAGGAACG |
| 1198 | DR-VASN for | GTCACCGGCCCTACATCTAAGCCAGAGAGAT |
| 1199 | DR-VASN rev | CCGGATCTCTCTGGCTTAGATGTAGGGCCG |
| 1202 | HA-myc LDHB for | GCGCGCTAGCATGTACCCATACGATGTTCCAGATTACGCTGCAACTCTTAAGGAAAAACTC |
| 1203 | HA-myc LDHB rev | GCGCGGATCCCAGCCTAGAGCTCACTAG |
| 1229 | DR-VASN-dTAA for | GTCACCGGCCCTACATCTAATAAAGAGAGAT |
| 1230 | DR-VASN-dTAA rev | CCGGATCTCTCTTTATTAGATGTAGGGCCG |
| JH59 | DR-AQP4 for | GTCACCGGTCTTCAGTATGACTAGAAGATCT |
| JH60 | DR-AQP4 rev | CCGGAGATCTTCTAGTCATACTGAAGACCG |
| JH61 | DR-SYTL2 for | GTCACCGCTCAGCCTCCTGACTAGCTGGAAT |
| JH62 | DR-SYTL2 rev | CCGGATTCCAGCTAGTCAGGAGGCTGAGCG |
| JH67 | DR-CACNA2D4 for | GTCACCGTGACTATGTATGACTATCAGGCCT |
| JH68 | DR-CACNA2D4 rev | CCGGAGGCCTGATAGTCATACATAGTCACG |
| JH81 | DR-DHX38 for | GTCACCGGGTGTATGCGTGACTTGGCTGTGT |
| JH82 | DR-DHX38 rev | CCGGACACAGCCAAGTCACGCATACACCCG |
